# Chiropractic care and research priorities for the pediatric population: a cross-sectional survey of Quebec chiropractors

**DOI:** 10.1186/s12998-023-00514-z

**Published:** 2023-09-26

**Authors:** Rebecca Hayes, Camille Imbeau, Katherine A Pohlman, Marc-André Blanchette, Chantale Doucet

**Affiliations:** 1 Département de Chiropratique, Université du Québec à Trois-Rivières (UQTR), 3351, Boul. Des Forges, C.P. 500, Trois-Rivières, QC G9A 5H7 Canada; 2 Research Center, Parker University, Dallas, TX USA

**Keywords:** Research, Pediatric care, Treatment modalities

## Abstract

**Background:**

Chiropractors commonly treat pediatric patients within their private practices. The objectives of this study were (1) to identify the treatment techniques and health advice used by Quebec chiropractors with pediatric patients; (2) to explore the research priorities of Quebec chiropractors for the pediatric population; and (3) to identify Quebec chiropractors’ training in the field of pediatric chiropractics.

**Methods:**

A web-based cross-sectional survey was conducted among all licensed Quebec chiropractors (Qc, Canada). Descriptive statistics were used to analyze all quantitative variables.

**Results:**

The results showed that among the 245 respondents (22.8% response rate), practitioners adapted their treatment techniques based on their patients’ age group, thus using softer techniques with younger pediatric patients and slowly gravitating toward techniques used with adults when patients reached the age of six. In terms of continuing education, chiropractors reported an average of 7.87 h of training on the subject per year, which mostly came from either Quebec’s College of Chiropractors (OCQ) (54.7%), written articles (46.9%) or seminars and conferences (43.7%). Both musculoskeletal (MSK) and viscerosomatic conditions were identified as high research priorities by the clinicians.

**Conclusions:**

Quebec chiropractors adapt their treatment techniques to pediatric patients. In light of limited sources of continuing education in the field of pediatric chiropractics, practitioners mostly rely on the training provided by their provincial college and scientific publications. According to practitioners, future research priorities for pediatric care should focus on both MSK conditions and non-MSK conditions.

## Background

Worldwide, the lifetime utilization rate of chiropractic care is approximately 22.2% (IQR: 12.8–40.0). Although the prevalence of children younger than 18 seeking chiropractic care is lower than that in the adult population, globally, in the pediatric population, the estimated prevalence of use of chiropractic care over 12 months is 8.1% (IQR: 3.8–20.0), and the lifetime utilization rate is 11.1% (IQR: 4.0-21.6) [1]. Among pediatric patients in Quebec seeking care in chiropractic clinics, adolescents are the most common, followed by infants, school-age children and toddlers [2]. Musculoskeletal (MSK) conditions, excessive crying, neurological conditions and gastrointestinal conditions are stated as the main reasons for seeking chiropractic care in the pediatric population; however, other MSK or non-MSK conditions (ear, nose and throat conditions, infection, headache, asthma, stomach conditions, etc.) are also stated as less frequent reasons for children to attend chiropractic visits [1].

In recent years, literature reviews have been conducted to inform decision making related to pediatric chiropractic care [3–5]. Although the reviews found little to no evidence of pediatric patient harm by spinal manipulation [4, 5], they highlight the lack of strong scientific data and evidence in the field [1, 3]. Consequently, it is of great importance for the chiropractic profession to gather information on the types of treatments provided and the sources of information used by professionals to establish research priorities to ensure the development of knowledge in the field.

As a first step toward contributing to the development of knowledge in the field of pediatric chiropractics, the objectives of this study are (1) to identify the treatment techniques and health advice used by Quebec chiropractors with pediatric patients, (2) to explore the research priorities of Quebec chiropractors for the pediatric population, and (3) to identify Quebec chiropractors’ training in the field of pediatric chiropractics.

## Methods

### Ethical considerations

The ethics board for research involving humans of the Université du Québec à Trois-Rivières (UQTR) determined that ethical certification was not needed. Participation was voluntary, and the participants provided consent virtually prior to completing our survey.

### Study design and participants

We conducted a web-based cross-sectional survey that was sent via email to all members of the “Ordre des Chiropraticiens du Québec” (OCQ) starting in July 2019 [6]. In the province of Quebec, only members of the OCQ are qualified to practice chiropractics, and most of them have their professional contact information listed on the OCQ website. Respondents with less than one year of experience, those who did not treat the pediatric population and those without active practice in Quebec were excluded from the study to capture a meaningful portrait of chiropractic pediatric care in Quebec.

### Survey instrument

The survey questionnaire was adapted from a previous Swiss survey on the same topic [7]. Questions regarding the ranking of future research priorities, advice commonly given to pediatric patients and sources of information considered for pediatric care were added to the questionnaire by the investigators for the purpose of this study. The complete questionnaire is provided in a previous publication [2]. Briefly, a pilot study was carried out with a convenience sample of 29 chiropractors (May-June 2019) recruited on social media via posts on two Facebook pages targeting Quebec chiropractors with pediatric interests [8]. Respondents were invited to provide any type of feedback they deemed appropriate to improve the clarity and comprehensiveness of the questionnaire.

#### Description of the study variables

##### Pediatric care

Participants were asked to select the treatment techniques they used in each pediatric age group (0–6 months, 7–23 months, 2–5 years, 6–12 years,13–17) from the following eleven options: high-velocity low-amplitude adjustment, low-velocity low-amplitude adjustment, instrument-assisted (Activator, Arthrostim, etc.) techniques, use of a speeder board for the cervical region, cranial techniques, nonsegmental techniques, upper cervical techniques, soft tissue mobilizations, kinesio-taping, soft tissue therapy and other. They were also asked to estimate at which frequency (never, rarely [1x/4 weeks], few times [1x/2 weeks], often [2x/week, frequently [5x/week]) they provided health advice, such as breast feeding advice, advice on changing unhealthy or risky habits, prevention or screening advice, nutritional recommendations, recommendation of physical activity, relaxation or stress reduction advice and strategies for at-home care, to their pediatric patients.

##### Continuous education

Respondents had to identify which of the continuing education courses in pediatrics listed in Table 1 they attended along with their perinatal organization membership and chiropractic treatment goal. The average number of continuing education hours they yearly completed in pediatrics was also requested.

**Table 1:** Characteristics of pediatrics chiropractic continuing education program (N = 245)

		N (%) or [Mean (SD)]
**Sources of continuing education in the field of chiropractic pediatrics N (%)**		
	OCQ	134 (54.7)
	Written articles	115 (46.9)
	Seminars / conferences	107 (43.7)
	Post-graduate courses	73 (29.8)
	AQCPP	63 (25.7)
	Other	24 (9.8)
	Pediatric hospital	3 (1.2)
**Average hours per year of continuing education in the field of chiropractic pediatrics [Mean (SD)]**		[8 (22)]
**Professional profiles of respondents (memberships to pediatric organisations)** **N (%)**		
	None	126 (50.4)
	AQCPP	54 (22.0)
	ICPA	36 (14.7)
	ICA	25 (10.2)
	MAQ	3 (1.2)
	Nourri-source	1 (0.4)
	Nourrison lait	1 (0.4)
	ACQ	1 (0.4)
**Main objective of chiropractic treatment** **N (%)**		
	Improve function	116 (47.3)
	Reduce pain	29 (11.8)
	Prevention	18 (7.3)
	Remove subluxations	25 (10.2)
	Improve lifestyle	1 (0.4)
	Improve quality of life	41 (16.7)
	Other	15 (6.1)

AQCPP: Association québécoise de chiropratique pédiatrique et en périnatalité [Quebec’s provincial association of pediatrics and perinatal]

OCQ: Ordre des chiropracticiens du Québec [Quebec’s provincial association]

ICPA: International Chiropractic Pediatric Association [International Chiropractic Pediatric Association]

ICA: International Chiropractors Association [International Chiropractors Association]

MAQ: Mouvement allaitement Québec [Quebec based breastfeeding organization]

##### Research priorities

Participants were asked to rank in, in order of importance, the axes of future research in pediatric chiropractics listed in Table 2. The respondents were also encouraged to formulate additional research priorities in an open-ended question.

**Table 2:** Future research priorities for pediatric chiropractic care ranked by the respondents [1 (most important) to 10 (least important)]. (N = 245)

Research priorities	Mean (SD)	Mode
MSK conditions on fonction and pain of the cervical region	3 (2)	1
Viscero-somatic conditions	5 (3)	1
Determinant factors of MSK conditions affecting neuromotor development	5 (3)	1
MSK conditions on function and pain of the thoracic region	5 (2)	2
MSK conditions on fonction and pain of the lumbar region	5 (2)	3
Clinical conditions of the lower and upper limbs	6 (2)	6
Research on sport themes	7 (3)	6
Postural research	6 (2)	7
MSK problems concerning breastfeeding	6 (3)	9
Lifestyle research	7 (3)	10

MSK: Musculoskeletal

SD : Standard Deviation

### Data collection

The survey was administered using the *Banque interactive de question* (BIQ), a survey tool created by the *Université du Québec à Trois-Rivières* (https://confluence.uqtr.ca/display/AOPSP/BIQ). The data collection was interrupted from the first wave of the COVID-19 pandemic. In May 2021, the list of OCQ members was updated, and data collection resumed. In addition to the email invitations, social media posts were made on the two Facebook pages targeting chiropractors with pediatric interest in Quebec. Up to three weekly email reminders were sent to the nonresponders. The partial responders were contacted by phone by a research assistant to seek missing information. Data collection was completed in June 2021.

### Data analysis

Descriptive statistics (frequencies, proportions, mean, mode and standard deviation [SD]) were calculated for all quantitative variables. The test-retest reliability of our survey items was assessed using the percentage of agreement [8, 9]. All analyses were conducted in SPSS for Mac (version 28.0; IBM Corporation, Armonk, NY). The qualitative data extracted from the practitioners’ suggestions for future research priority subjects were divided into themes by the main author using content analysis (MSK conditions, non-MSK conditions, general health screening and others) [10]. The classification generated was then revised by a senior researcher (MAB).

## Results

### Study population

Among the 1286 registered members of the OCQ in 2021, 1175 chiropractors (91.4%) had publicly available email information and were invited to complete our survey. Two hundred sixty-one respondents (22.2%) completed our survey, but sixteen were excluded because they did not meet the inclusion criteria (7 did not practice in QC, 8 did not treat the pediatric population and 1 had been in practice for less than 12 months). The analyzed sample included 245 respondents, for an effective response rate of 20.9%. Based on our comparison of the sample data with the available data for the complete membership of the OCQ, our sample presented a similar number of years of experience but included significantly more women (62.9% compared to 46.0%) [2].

### Pediatric care/continuous education

The amount and sources of continuing education in the field of chiropractic pediatrics are presented in Table 1 along with the pediatric organization membership and treatment objectives of our respondents. On average, the respondents reported that eight hours of their continuing education per year was related to pediatric care. The most common source of training was the OCQ (Quebec’s Provincial College of Chiropractors) (54.7%) and written articles (46.9%). The most popular pediatric organization was the Provincial Association for Chiropractic Pediatric Care (22,0%), but the majority of the respondents did not report any pediatric organization membership (50.4%). The respondents’ most common treatment objective was to improve function (47.3%).

The treatment modalities used for each pediatric age group are presented in Table 3. The majority of the respondents reported using instrument-assisted spinal manipulations, soft tissue mobilizations and therapies in all age groups. Low-velocity low-amplitude spinal manipulations and cranial techniques were reported by the majority of the respondents for children younger than 2 years. High-velocity low-amplitude spinal manipulations were reported by the majority of the respondents for children older than 2 years.

**Table 3:** Treatment techniques/modalities used by pediatric age groups (N = 245)

Treatment techniques, N (%)	0–6 months	7–23 months	2–5 years	6–12 years	13–17 years
High velocity low amplitude adjustment	40 (16.3)	66 (26.9)	147 (60.0)	**193 (78.8)**	**217 (88.6)**
Low velocity low amplitude adjustment	136 (55.5)	140 (57.1)	117 (47.8)	105 (42.9)	95 (38.8)
Instrument-assisted (Activator, Arthrostim, etc.)	143 (58.4)	156 (63.7)	**165 (67.3)**	171 (69.8)	161 (65.7)
Speader board for cervical region	102 (41.6)	33 (13.5)	0 (0.0)	0 (0.0)	0 (0.0)
Cranial techniques	**153 (62.4)**	147 (60.0)	116 (47.3)	107 (43.7)	99 (40.4)
Non-segmental techniques	10 (4.1)	16 (6.5)	16 (6.5)	19 (7.8)	23 (9.4)
Upper cervical technique	15 (6.1)	16 (6.5)	21 (8.6)	27 (11.0)	27 (11.0)
Soft tissue mobilizations	139 (56.7)	**143 (58.4)**	154 (62.9)	160 (65.3)	162 (66.1)
Kinesio-taping	37 (15.1)	43 (17.6)	58 (23.7)	110 (44.9)	133 (54.3)
Soft tissue therapy	121 (49.4)	138 (56.3)	154 (62.9)	177 (72.2)	186 (75.9)
Other	29 (11.8)	22 (9.0)	21 (8.6)	18 (7.3)	22 (9.0)

Mode: **Bold**

The frequency of health advice provided to the pediatric population by age group is presented in Table 4. The majority of the health advice was provided once or twice a month, except for advice promoting physical activity and strategies for homecare, which were provided on a weekly basis.

**Table 4:** Frequency of health advice provided by chiropractors to pediatric patients (N = 245)

Health advices, N (%)	Never	Rarely (1x/4weeks)	Few times (1x/2weeks)	Often (2x/week)	Frequently (5x/week)	Missing
Breast feeding advice	69 (28.2)	**90 (36.7)**	49 (20.0)	24 (9.8)	12 (4.9)	1 (0.4)
Changing unhealthy or risky habits	19 (7.8)	57 (23.3)	**74 (30.2)**	55 (22.4)	38 (15.5)	2 (0.8)
Prevention or screening purposes	12 (4.9)	**87 (53.5)**	82 (33.5)	45 (18.4)	17 (6.9)	2 (0.8)
Nutritional recommendations	42 (17.1)	**88 (35.9)**	50 (20.4)	45 (18.4)	19 (7.8)	1 (0.4)
Promoting physical activity	8 (3.3)	36 (14.7)	46 (18.8)	74 (30.2)	**79 (32.2)**	2 (0.8)
Relaxation or stress reduction	20 (8.20	**65 (26.5)**	49 (20.0)	61 (24.9)	48 (19.6)	2 (0.8)
Strategies for at home care	25 (10.2)	47 (19.2)	48 (19.6)	**66 (26.9)**	57 (23.3)	2 (0.8)

Mode: **Bold**

### Research priorities

The mean ranks for each of the prespecified research priorities are presented in Table 2. MSK conditions affecting function and pain of the cervical region, viscerosomatic conditions and the determinant factors of MSK conditions affecting neuromotor development were the top three research priorities from the respondents’ perspective.

The main themes emerging from the research priorities suggested by the respondents in the open-ended question are reported in Table 5. The different themes are grouped into four categories: MSK conditions, non-MSK conditions, general health screening and others.

**Table 5:** Themes emerging from the open ended suggestions for future research priorities for pediatric chiropractic care (145 respondents provided an answer)

**MSK CONDITIONS**
Pediatric postural anomalies
Pediatric developmental anomalies and conditions (MSK)
Pediatric growth pain
Pediatric spinal pain
Headaches
**NON-MSK CONDITIONS**
Pediatric developmental anomalies and conditions (neuro-psycho-immune)
Pediatric otolaryngological conditions
Pediatric viscero-somatic conditions
Post pediatric stroke follow-up
Childbirth and physiology of the pregnant woman
**GENERAL HEALTH SCREENING**
Mental health disorders
Nutritional advice for parents and children
Pediatric neurodevelopment disorders
Child sexual abuse
Vaccines
**OTHERS**
Representation of the profession and pediatric practice amoung Quebec chiropractors
Safety of chiropractic pediatric care
Efficiency of preventive chiropractic pediatric care

MSK: Musculoskeletal

## Discussion

As illustrated in a previous publication on this descriptive cross-sectional study, Quebec chiropractors commonly provide pediatric care but do not exclusively treat pediatric populations [2]. Our results reveal that chiropractors adapt their treatment techniques based on the patient’s age, using softer techniques on younger patients and slowly gravitating toward more commonly used techniques on adults when patients reach the age of six years. This appears to be consistent with a recent publication that showed differences in spine tensile strength between adult and pediatric patients and that proposed a preliminary model of care in which it is recommended that spinal manipulation therapy (SMT) techniques be adapted based on the patient’s age [11].

As this study has shown, a small to moderate percentage of practitioners use high-velocity low-amplitude techniques and techniques assisted by instruments, such as activators, to treat pediatric age groups. Based on a survey of chiropractors, infants aged 0 to 2 months old should receive only 10% of the equivalent adult SMT force (roughly 2 N), and toddlers aged 3–23 months should receive approximately 30% of the adult SMT force (5.8 N) [11, 12]. Research on adverse events and SMT in children has not demonstrated a difference between instrument-assisted techniques and manipulation [13]. More research on the thresholds of SMT force for children and adverse events are needed to draw any conclusions about this finding.

Common therapeutic objectives of the respondents in this study were to improve function (47.3%) and improve quality of life (16.7%). A minority of the respondents were focused on removing subluxations (10.2%), which might reflect a vitalistic or more conservative philosophy based on the traditional chiropractic paradigm [14]. This faction in the profession is likely to retain opinions and perspectives that are in contrast with current scientific paradigms, as illustrated in a previous publication [15]. Adhering to the vitalistic paradigm has previously been identified as a barrier to interprofessional collaboration [16].

The chiropractors in this study mainly promoted physical activity and strategies for at-home care when giving health advice to patients, while they scarcely included nutritional recommendations or advice on the prevention or screening of disease. Nutritional recommendations are currently given by practitioners but are not included within the scope of MSK recommendations and might not be aligned with current best practice and recommendations [17]. If chiropractors were to provide wider public and preventative health advice to patients, future research on the matter would be recommended.

### Continuing education

Although Quebec chiropractors see pediatric patients for a variety of health conditions, the respondents in this study considered their undergraduate training on the subject to be suboptimal; they believed that educational content included in the core curriculum should be augmented and developed in collaboration with institutions/organizations with expertise in the matter and then included in the core curriculum [18]. This study showed that most chiropractors had approximately 8 h of continuing education in the field of chiropractic pediatrics per year that mostly came from either the OCQ, written articles or seminars and conferences. Most Quebec chiropractors were not part of any pediatric organizations, but those who were members of such organizations were linked to either the *Association québécoise de chiropratique pédiatrique et en périnatalité* (AQCPP), International Chiropractors Association (ICA) or International Chiropractic Pediatric Association (IPCA). The main source cited for continuing education was the OCQ. An examination of the past yearly continuous education programs offered by the OCQ indicated that the last educational conferences on the subject were in 2017. Hence, the respondents in this study might have been influenced by the OCQ’s conference agenda that year. Since then, no further pediatric content has been included in their program. Written articles could be an excellent source of information, but it is unclear which articles chiropractors are reading and how the data extracted from them translate into day-to-day practice. Furthermore, the current scientific evidence on the topic of pediatric chiropractics remains limited, and it has been stated that more high-quality research and clinical trials are necessary [3]. Finally, seminars and conferences on the subject of pediatric chiropractic tend to be very rare and are not necessarily given by a licensed organization with clear, evidence-based competencies [19], which prevents them from being a valid source of training. More broadly, although some continuing education in the field is obtained by the doctors, the source and accuracy of their information is unclear due to the lack of current high-quality scientific data available in the field [20].

### Research priorities

This is the first Canadian study identifying research priorities for chiropractic pediatric care from the clinician perspective. When asked to rank future research focuses in the field of pediatric chiropractics, the respondents mainly focused on the subjects of effectiveness of chiropractics for MSK and viscerosomatic conditions. In comparison, it was previously shown that Canadian chiropractic organizations had identified priorities in the area of health services, such as the integration of chiropractic care into multidisciplinary settings, the cost-effectiveness of chiropractic care and the effect of chiropractic care on reducing medical services [21].

In studies across Europe and Canada, research priorities such as the cost-effectiveness of chiropractic care, as well as multidisciplinary and interprofessional collaboration, were recurrent topics among academic members or organizations of the profession [21, 22]. These results show a gap between practitioners’ and the priorities in future research agendas, thus going against previous research that showed an overall agreement between practitioners and academics across most research priorities [23]. It seems that organizations tend to prioritize subjects in the area of health services, while practitioners tend to gravitate toward the effectiveness of chiropractic treatment on different health issues with the hopes of obtaining data on chiropractic treatment outcomes for conditions for which there is sometimes a lack of scientific evidence.

Furthermore, the respondents in this study rated viscerosomatic conditions as one of their two main future research focuses in the field of pediatric chiropractic, even though the conclusions of recent systematic reviews on the effectiveness of manual therapy on some non-MSK conditions showed no evidence of an effect [24, 25]. The interest behind the treatment of non-MSK conditions might be related to the fact that recent systematic reviews have not been based on topics or conditions of interest for clinicians. In light of our findings, although chiropractors are trained and considered MSK experts, some practitioners may have higher expectations in terms of their ability to treat non-MSK conditions with the pediatric population than what recent literature on the subject suggests.

### Limitations

Our findings might have limited generalizability given our response rate of 21% and the higher proportion of women in the sample than in the Quebec chiropractor population; however, a higher proportion of women than men may be attending to the pediatric population, as shown in the medical profession [26]. It is likely that the respondents in this study might have had a specific interest in pediatric care since 22% of them reported being a part of the AQCPP, an association that includes only 11% of Quebec chiropractors. Although our questionnaire demonstrated satisfactory reliability, its validity was not assessed. Therefore, it is possible that the responses provided by the respondents might not have perfectly reflected reality. For the specific question on research priorities, the scope of the assessed priorities might have been limited because the question started with preestablished research categories; however, we attempted to resolve this issue with the use of an open-ended question, which was completed by 59% of the respondents. Furthermore, it is unclear how the COVID-19 pandemic might have influenced our data collection.

## Conclusion

This study that evaluated Quebec chiropractor care of the pediatric population found that these chiropractors adapted their treatment techniques based on their pediatric patients’ age with high-velocity low-amplitude/instrument-assisted treatments being used for all age groups. In light of limited sources of continuing education in the field of pediatric chiropractics within Quebec, practitioners mostly turned to their own professional organizations for training. Based on the practitioners’ opinions, future research priorities should focus not only on MSK conditions but also on non-MSK conditions within the pediatric population, for which chiropractics has not yet been shown to have any effect.

## Data Availability

The datasets used and/or analysed during the current study are available from the corresponding author on reasonable request.

## List of abbreviations

MSK: Muskuloskeletal
SMT: Spinal Manipulation Therapy
AQCPP: Association québécoise de chiropratique pédiatrique et en périnatalité
ICA: International chiropractors association
IPCA: International chiropractic pediatric association
OCQ: Ordre des Chiropraticiens du Québec
